# Food Allergies: The Basics

**DOI:** 10.1053/j.gastro.2015.02.006

**Published:** 2015-05

**Authors:** Rudolf Valenta, Heidrun Hochwallner, Birgit Linhart, Sandra Pahr

**Affiliations:** Division of Immunopathology, Department of Pathophysiology and Allergy Research, Center for Pathophysiology, Infectiology and Immunology, Medical University of Vienna, Vienna, Austria

**Keywords:** IgE-Associated Food Allergy, Allergen, IgE, Diagnosis, Multiallergen Test, Therapy, Immunotherapy, FcεRI, Fc epsilon receptor I, IL, interleukin, OAS, oral allergy syndrome, SIgA, secretory IgA, SIT, specific immunotherapy, Th, T-helper

## Abstract

IgE-associated food allergy affects approximately 3% of the population and has severe effects on the daily life of patients—manifestations occur not only in the gastrointestinal tract but also affect other organ systems. Birth cohort studies have shown that allergic sensitization to food allergens develops early in childhood. Mechanisms of pathogenesis include cross-linking of mast cell– and basophil-bound IgE and immediate release of inflammatory mediators, as well as late-phase and chronic allergic inflammation, resulting from T-cell, basophil, and eosinophil activation. Researchers have begun to characterize the molecular features of food allergens and have developed chip-based assays for multiple allergens. These have provided information about cross-reactivity among different sources of food allergens, identified disease-causing food allergens, and helped us to estimate the severity and types of allergic reactions in patients. Importantly, learning about the structure of disease-causing food allergens has allowed researchers to engineer synthetic and recombinant vaccines.

**Figure:**
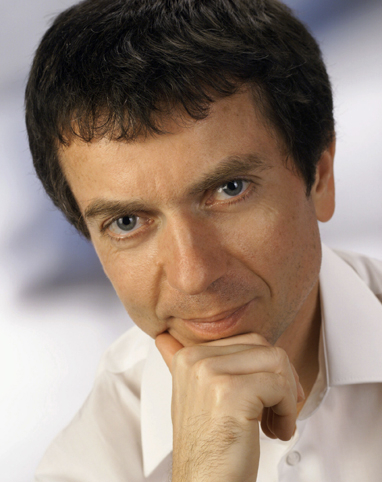
Rudolf Valenta

**Figure:**
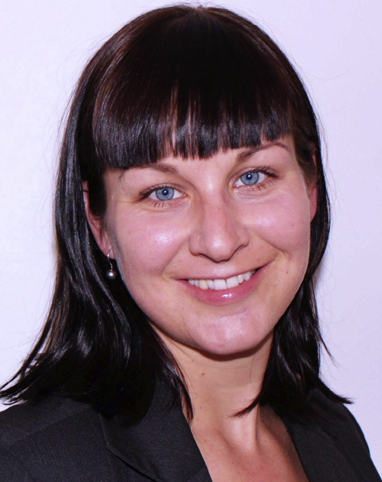
Heidrun Hochwallner

**Figure:**
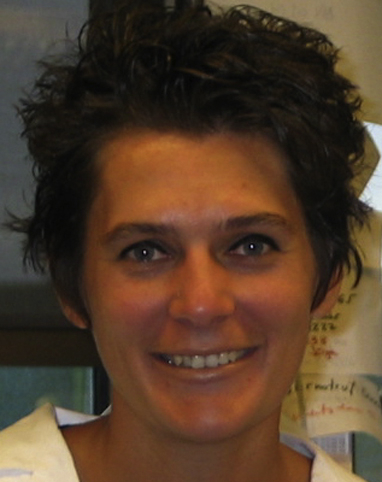
Birgit Linhart

**Figure:**
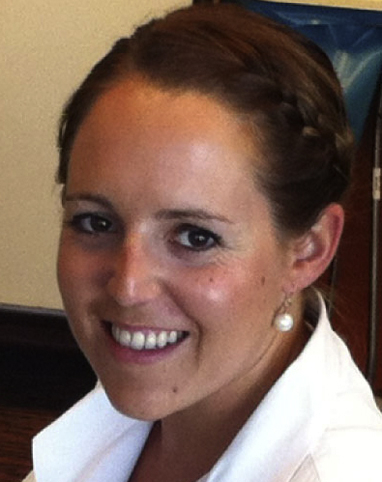
Sandra Pahr

There are several mechanisms by which people develop adverse reactions to foods also termed *food intolerance*.^1^ These reactions can be considered toxic or nontoxic (Figure 1).^2^ Among the nontoxic reactions, those that are not immune-mediated, such as those involving enzyme defects (eg, vasoactive amines) or reactions to certain substances (eg lactose intolerance), are far more common than immune-mediated reactions.^2^ Nevertheless, immune-mediated reactions affect millions of people, are responsible for significant morbidity and health care costs, and can cause severe life-threatening reactions that lead to death.^3^, ^4^, ^5^ Food allergy was defined by an expert panel of the National Institute of Allergy and Infectious Diseases as “an adverse health effect arising from a specific immune response that occurs reproducibly on exposure to a given food.” This response comprises basically all types of immune-mediated reactions, including those caused by the adaptive and innate immune system (Figure 1).^6^

**Figure 1:**
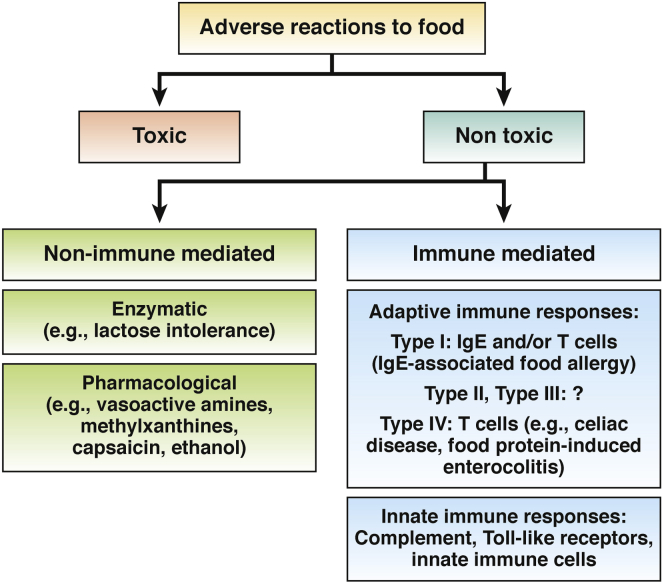
Classification of food intolerance. Adverse reactions to food can be classified as toxic or nontoxic reactions. Nontoxic reactions are categorized further as immune-mediated or non–immune-mediated. The most common adverse reactions are based on non–immune-mediated mechanisms such as enzyme defects as observed in lactose intolerance. Hypersensitivities involving the adaptive immune system can be subdivided into 4 categories (types I–IV). Type I reactions are always associated with the formation of IgE against food allergens and therefore can be called IgE-associated food allergies. There is firm evidence for an involvement of IgG in type II or type III reactions in immune-mediated adverse reactions to food, whereas type IV reactions, which involve T cells, have important roles in disorders such as celiac disease. There is evidence that the innate immune system, which includes complement, Toll-like receptors, and innate immune cells, also mediates immune reactions against certain food components.

The term *allergy* was coined in 1906 by the Austrian pediatrician Clemens von Pirquet,^7^ who described cases of serum sickness in children treated with antibody preparations. According to Coombs and Gell,^8^ there are 4 major types of allergic reactions based on pathogenesis mechanisms. The most common forms of immune-mediated adverse reactions to foods (type I reactions) always are characterized by the development of IgE against food allergens. It can be accompanied by inflammation, induced by cellular components, and mediated by T cells and eosinophils. Patients with IgE-associated food allergy can be identified based on the detection of food allergen–specific IgE in serum and body fluids, and by measuring IgE-mediated cellular and in vivo responses.^4^

Although it is tempting to speculate that food antigen–specific IgG can cause adverse reactions via type II or type III hypersensitivity, there is no solid experimental evidence to support the relevance of these reactions to food allergies that develop in patients (Figure 1). Accordingly, several position papers strongly recommend against testing for food antigen–specific IgG in the diagnosis of food allergy.^9^, ^10^

Type IV hypersensitivity, which mainly involves food antigen–specific T-cell responses and can damage the gut mucosa, is associated with disorders such as celiac disease. Celiac disease is characterized by a hypersensitivity reaction against the wheat gluten fraction comprising alcohol soluble gliadins and acid-, alkali-soluble glutenins, accompanied by an autoimmune component.^11^ Type IV hypersensitivity reactions also might be involved in food protein–induced enterocolitis (Figure 1).^12^ Studies have shown that certain food proteins can induce inflammation via direct activation of the innate immune system. For example, wheat amylase trypsin inhibitors and certain milk oligosaccharides can cause intestinal inflammation via activation of Toll-like receptor 4,^13^, ^14^ and certain allergens have been shown to stimulate the innate immune system.^15^ Innate immune mechanisms might mediate nonceliac gluten sensitivity.^16^

In developed countries, IgE-associated food allergy affects 3%–8% of children and 1%–3% of adults.^3^, ^4^, ^5^ It not only is common, but often is a serious and life-threatening health condition that requires accurate diagnosis and has strong effects on an individual’s dietary habits and social life. Milk, eggs, wheat, peanuts, nuts, sesame, fish, fruits, and vegetables are common inducers of IgE-associated food allergy.^4^ Allergies to foods such as milk, egg, and wheat often are outgrown (patients acquire tolerance), whereas allergies to peanuts, tree nuts, and fish allergies often persists over a lifetime.^3^ The exact incidence of food allergies has not been fully established because there are discrepancies among findings from studies in which food allergies were self-reported vs those diagnosed by various assays (eg, provocation, skin test, or serologic tests).^4^, ^17^

The prevalence and severity of food allergies seem to be increasing. In addition to genetic factors, a number of environmental, cultural, and behavioral factors affect the frequency, severity, and type of allergic manifestations in patients.^18^, ^19^, ^20^, ^21^ A recent study identified epigenetic differences in CD4+ T cells from children with IgE-mediated food allergies, compared with children without food allergies—differences such as these might contribute to the development of a food allergy.^22^ According to the hygiene hypothesis, decreases in family size and improvements in personal hygiene have contributed to the increased prevalence of IgE-mediated allergies. On the other hand, factors such as an anthroposophic lifestyle (eating organic foods that contain lactobacilli and restrictive use of antibiotics, antipyretics, and vaccines) have been associated with a reduced incidence of allergies.^23^, ^24^ It has been proposed that insufficient exposure to dietary and bacterial metabolites might have contributed to increases in inflammatory disorders in Western countries.^25^

## Allergic Sensitization and Secondary Immune Responses

**Figure 2:**
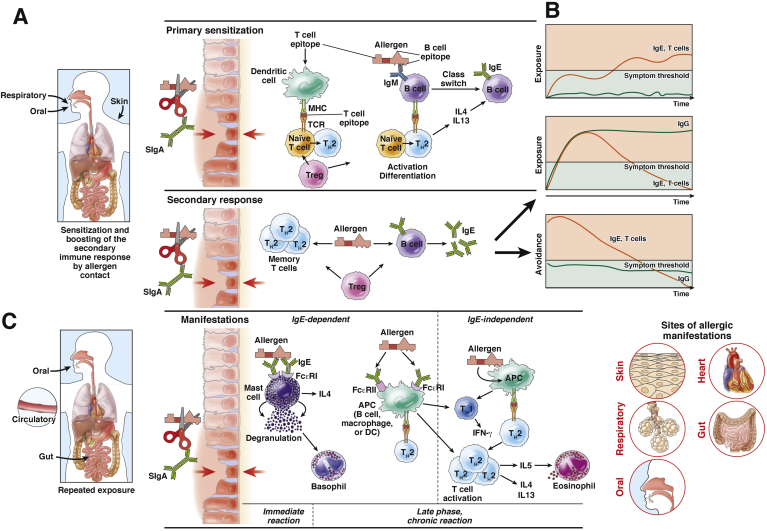
Time course, pathogenesis, and manifestations of food allergies. IgE-associated food allergies appear to develop early in childhood. This process is termed *allergic sensitization*. (*A*) Allergen contact via the gastrointestinal tract, via the respiratory tract, and eventually via the skin induces IgE production (primary sensitization) in genetically predisposed individuals. Repeated allergen contact activates allergen-specific T cells and induces IgE responses during the secondary immune response. Factors that affect the epithelial barrier (*red arrows*) and the extent to which allergens are digested or degraded are important for primary sensitization and boosting of secondary immune responses. SIgA and T-regulatory cells may be important for exclusion of allergens from the intestinal lumen and induction of tolerance, respectively. (*B*) The balance between allergen-specific IgE and blocking IgG helps determine whether or not a patient will develop symptoms. Allergen avoidance could reduce levels of allergen-specific IgE to below the threshold for symptom induction (*lower panel*), whereas exposure could increase production of IgE, leading to symptoms (*upper panel*). If allergen exposure induces allergen-specific IgG, which blocks the interaction between the allergen and IgE, then symptoms might be reduced (*middle panel*). (*C*) Allergy symptoms are caused by repeated contact with the oral allergen, via the immediate allergic reaction (allergen-induced cross-linking of mast cell–bound IgE by allergen and then activation of allergen-specific T cells), and then by other inflammatory cells, such as eosinophils and basophils, during late-phase and chronic inflammation. Factors that affect the epithelial barrier and the extent of allergen degradation affect the amount of allergen intrusion and the magnitude and type of inflammation. After allergen ingestion, inflammation develops not only in the intestine, but in other organs, such as the skin, respiratory tract, and circulatory system (*right*). These allergens and allergen fragments are internalized and distributed throughout the body (*left*). MHC, major histocompatibility complex; T-reg, T-regulatory cell; TCR, T-cell receptor.

The term *allergic sensitization* describes the first induction of an allergic immune response upon allergen encounter.^26^, ^27^ Two routes of allergic sensitization are well established (Figure 2*A*). Class 1 food allergens (eg, milk, egg, or peanut) are oral allergens that cause sensitization via the gastrointestinal tract.^28^ Class 2 food allergens are aeroallergens (eg, major birch pollen allergen Bet v 1) that cause sensitization via the respiratory tract. Immune responses against these allergens can cross-react with homologous food allergens (eg, major apple allergen Mal d 1) to cause symptoms.^29^, ^30^, ^31^

It recently was proposed that people become sensitized to food allergens via skin contact, but there have been few studies of this process.^18^ Interestingly, studies of animal models have indicated that epicutaneous sensitization leads to expansion of IgE-dependent intestinal mast cells and food-induced allergic reactions.^32^, ^33^ For an overview of food allergen sources that may cause sensitization via the respiratory tract and skin, see the article by Asero and Antonicelli.^34^

Determinants of allergic sensitization include features of the epithelial barrier, the allergen itself (whether allergens are stable and not degraded in the environment or gastrointestinal tract), nonallergenic components of the food matrix, and substances that act as adjuvants (Figure 2*A*).^35^ For example, food allergens have been proposed to have greater stability during digestion than other molecules in food.^36^ Intrinsic factors (eg, genetic factors such as mutations in the *filaggrin* gene) and exogenous factors (eg, alcohol, anti-inflammatory drugs, pathogens, or stress) have been proposed to reduce the barrier function of the intestinal epithelium and facilitate sensitization.^37^, ^38^, ^39^ On the other hand, secretory antibodies, particularly secretory IgA (SIgA), have important roles in reinforcing the epithelial barrier. Mice deficient in SIgA and secretory IgM are prone to develop food allergen–induced anaphylactic shock, which can be overcome by induction of tolerance with T-regulatory cells.^40^, ^41^

Many environmental and genetic factors contribute to the atopic predisposition of individuals. These determine their susceptibility to develop allergic immune responses against allergens.^42^ In atopic individuals who have a predisposition toward developing IgE-associated allergies, encounters with allergen activate, after processing by antigen-presenting cells (eg, dendritic cells or B cells), allergen-specific T-helper 2 (Th2) cells, which produce cytokines such as interleukin (IL)4 and IL13. These cytokines induce class switching and production of allergen-specific IgE.^43^, ^44^, ^45^ Primary allergic sensitization (such as a class switch toward IgE production) occurs early in life and leads to T-cell and IgE memory, which can be boosted with repeated allergen contact (secondary immune response).^46^, ^47^, ^48^, ^49^ Upon contact with a primary food allergen, nonallergic individuals produce allergen-specific IgG and IgA, which do not induce allergic reactions. The formation of food allergen–specific IgE is a main feature of IgE-associated food allergy and its diagnosis.

Analyses of the time courses of allergic sensitization to respiratory and food allergen sources in large birth cohort studies have shown that food allergies and their associated symptoms develop before respiratory allergies.^50^ In later life, there is a reverse trend—food allergies often are outgrown and respiratory allergies increase and dominate.^50^ Interestingly, the prevalence of food allergies is approximately 10-fold lower than that of respiratory allergies.^4^, ^51^ This could be because oral exposure to allergens activates tolerance mechanisms (via regulatory T cells) and less frequently results in allergic sensitization than respiratory exposure to allergens.^52^, ^53^

Several cellular mechanisms seem to influence primary allergic sensitization vs tolerance in the intestine. Tolerance can be mediated by antigen presentation by dendritic cells, which interact with C-type lectin receptors^54^; dendritic cell–bound IgE can down-regulate allergic inflammation at mucosal sites.^55^ Children with an egg allergy were reported to have reduced function of neonatal T-regulatory cells compared with children without an egg allergy.^56^ On the other hand, children who outgrew a cow’s milk allergy had increased T-regulatory cell responses.^57^ These findings indicate that T-regulatory cells modulate the development of food allergies.^58^

After primary sensitization, the allergic immune response is boosted with repeated exposure to allergen, increasing activation of allergen-specific T cells and production of IgE.

In persons with a respiratory allergy, the IgE response is boosted by contact with a mucosal allergen^48^ and, interestingly, does not seem to require T-cell help.^59^, ^60^, ^61^ Another interesting feature of the established secondary IgE response is that in adults with an allergy, the profile of allergens recognized by IgE does not change substantially, whereas it seems that young children can be sensitized to new allergens.^62^, ^63^ In the case of a respiratory allergy, allergen contact through the respiratory mucosa strongly boosts IgE production, but has little influence on the other classes of allergen-specific antibodies (eg, IgA or IgG).^48^ The responding B ε memory cells may reside in the respiratory mucosa or the adjacent lymphoid tissues,^64^ but little is known about the precise location of the cells involved in secondary IgE responses in allergic patients.^65^

The mechanisms by which food allergen–specific IgE responses are boosted in patients with food allergies are poorly understood. When food allergens were administered orally, patients had strong increases in the production of allergen-specific IgG, accompanied by an initial boost of IgE.^66^, ^67^, ^68^ These findings indicated that allergen ingestion can boost allergen-specific production of IgG as well as IgE. It is possible that oral allergens can boost production of disease-causing IgE, as well as that of potentially protective IgG; this might explain why elimination or continued intake of food allergens can benefit patients.^69^, ^70^

It is clear that avoidance of food allergens over a prolonged period of time reduces levels of allergen-specific IgE below the threshold level for symptoms (Figure 2*B*). However, there is controversy about whether intake of food allergens is beneficial. If food allergen intake mainly stimulates production of protective IgG antibodies, tolerance could be induced and allergen-specific IgE production could be reduced. However, insufficient IgG production could cause IgE levels to increase, leading to increased sensitivity and symptoms (Figure 2*B*). Several recent studies have shown that the induction of allergen-specific IgG antibodies, which block IgE recognition of food allergens, is associated with the successful immunotherapy for food allergy.^68^

## Pathogenesis and Manifestations of Food Allergy

Upon interaction with food antigens, IgE becomes cross-linked and binds to mast cells and basophils via the high-affinity receptor FcεRI (Figure 2*C*).^71^ This process activates these cells, leading to the release of granules that contain preformed inflammatory mediators (eg, histamine), as well as de novo synthesis and/or release of inflammatory mediators (eg, leukotrienes), proteases (eg, tryptase), inflammatory cytokines (eg, IL4), and chemotactic molecules. Mast cells and basophils are activated within a few minutes of IgE cross-linking, therefore this process it called an *immediate allergic reaction*; symptoms occur shortly after allergen contact.

Because food allergens enter the blood via the gastrointestinal tract, symptoms can develop directly at the sites of allergen contact (eg, mouth, esophagus, and/or intestine), or in other organs. Systemic reactions occur when allergens capable of cross-linking effector cell-bound IgE pass the barrier of the mucosa into the circulation (Figure 2*C*, right). Allergen uptake also may affect the circulatory and nervous systems.

Factors that contribute to the type and severity of reactions include the amount of ingested allergen, the stability of the allergen against digestion, and the permeability of the epithelial barrier (Figure 2*C*). The immediate allergic reaction leads to intense inflammation that can become life-threatening. The release of vasoactive mediators into the circulation can lead to vascular collapse and anaphylactic shock.^72^ Supplementary Table 1 summarizes the clinical manifestations of food allergies, the organ systems affected by IgE-mediated mast cell and basophil degranulation, and the clinical aspects of gastrointestinal food allergy.^3^, ^6^

Studies performed with well-defined reagents (eg, monoclonal IgE, in vitro cellular systems, defined allergens, and IgE epitopes) have shown that the degranulation of effector cells, and therefore the intensity of the immediate-type reaction, increases with the number of IgE epitopes on an allergen, high levels of allergen-specific IgE, and high-affinity allergen-specific IgE.^73^, ^74^, ^75^ The fact that high levels of allergen-specific IgE cause up-regulation of FcεRI on mast cells and basophils, and thereby a more dense loading of these cells with IgE, could account for the association between levels of allergen-specific IgE against stable food allergens and the severity of allergic reactions.^76^, ^77^, ^78^ In addition to the levels of FcεRI on mast cells, the number of intestinal mast cells and basophils and (probably related to mast cell numbers and activation) the levels of intestinal tryptase also seem to be related to the severity of reactions to food allergens.^79^, ^80^, ^81^ Interestingly, studies performed in animal models have shown that cytokines such as IL4 can induce expansion of intestinal mast cells.^82^

In addition to the immediate allergic reaction (the most frequent pathogenic mechanism of IgE-associated allergies), late-phase allergic reactions also occur after allergen contact; there are 2 types. The late-phase response to allergens has been studied in mainly cutaneous models, such as skin blister and skin chamber models. Several hours after allergen contact and the immediate reaction, there is an influx of basophils and eosinophils.^83^ This influx is steroid-sensitive and seems to involve granulocyte-macrophage colony-stimulating factor.^84^ Relatively little is known about the importance of late-phase reactions in food allergy, but it is tempting to speculate that they could be involved in food allergen–induced forms of eosinophilic gastroenteritis.^85^ In fact, data from experimental animal models have indicated that thymic stromal lymphopoetin–induced basophil responses promote eosinophilic esophagitis.^86^ Interestingly, it also has been shown that enteric eosinophils not only contribute to inflammation, but control dendritic cells to initiate primary Th2 cell–mediated immune responses, indicating a complex interaction among cells in food allergies.^87^

In addition to the late-phase responses, delayed-type reactions can occur 24–48 hours after allergen contact. These resemble features of a type IV hypersensitivity reaction, involving allergen-specific T cells. Allergen-specific T cells can be activated via IgE-dependent and IgE-independent pathways (Figure 2). In fact, in patients with allergies, antigen-presenting cells express FcεRI as well as the low-affinity receptor for IgE (FcεRII also known as CD23). The cells use this receptor for IgE-facilitated allergen presentation—a process found to be more effective for T-cell activation than allergen presentation without IgE.^88^, ^89^ Studies performed with allergen peptides that do not react with IgE and recombinant allergen derivatives showed that activation of allergen-specific T cells also can occur without IgE, and lead to delayed-type allergic reactions in patients.^90^, ^91^

Induction of atopic dermatitis by food allergens has been shown to require not only Th2 cells (and Th2 cytokines such as IL4, IL13, and IL5—a cytokine that activates eosinophils), but also Th1 cells, which mediated delayed allergic inflammation.^92^ Interferon-γ, secreted by allergen-specific Th1 cells, was shown to induce epithelial damage in a model of respiratory allergy.^93^

The immediate- and delayed-type allergic inflammation that occurs during IgE-associated food allergy has been studied extensively in patients with oral allergy syndrome (OAS) (Supplementary Table 1). OAS is caused by sensitization to respiratory allergens that structurally are similar to allergens in foods, leading to a cross-reactive immune response. The most common form of OAS develops with sensitization to the major birch pollen allergen, Bet v 1. In patients with this form of OAS, the immune response cross-reacts with allergens in plant-derived food such as apples, nuts, carrots, and celery.^94^ This leads to local allergy symptoms of the immediate type (local itching and swelling of the lips or tongue), caused by IgE-mediated mast cell degranulation.^95^ However, Bet v 1–related plant food allergens are digested by the gastrointestinal tract, therefore systemic reactions occur only in exceptional cases (such as after consumption of large amounts of the plant food allergens during exercise), and anaphylactic shock does not occur.^96^ Likewise, cooking destroys IgEs, but leaves peptides recognized by allergen-specific T cells intact.^97^ Ingestion of Bet v 1–related plant food allergens therefore can activate allergen-specific T cells by IgE-independent mechanisms, and induce late-phase and chronic allergic inflammation to cause disorders such as atopic dermatitis in sensitized patients.^98^

Information on the time until onset of allergic reactions after food allergen intake (minutes–hours vs hours–days) and allergy phenotype (eg, urticaria vs atopic dermatitis) can help to determine whether the symptoms involve immediate IgE-mediated mast cell or basophil activation, or late-phase or chronic allergic inflammation caused by T-cell or eosinophil activation. Then, it is possible to select the most appropriate therapy. For example, immediate reactions would be treated with antihistamines, antileukotrienes, epinephrine, mast cell stabilizers, or anti-IgE, whereas late-phase chronic inflammation would be treated with steroids or anti-IL5.

## Preventing Food Allergies

According to the current food allergy and anaphylaxis guidelines of the European Academy of Allergy and Clinical Immunology,^99^ there are no restrictions regarding diet for mothers during pregnancy and lactation. Exclusive breastfeeding is recommended for the first 4–6 months of life, which prevents the development of allergies. If breastfeeding is not possible, hypoallergenic formulas with documented preventive effects are recommended for high-risk children. Breastfeeding transfers protective SIgA to the child, which may prevent allergic sensitization, and avoids early exposure to potential food allergens. This process may involve the uptake of SIgA-allergen complexes via receptors on M cells. Although certain studies have shown that early feeding of probiotics can reduce the development of allergic manifestations, particularly eczema, other studies have found that probiotic supplementation during early childhood did not prevent the development of allergic diseases.^100^, ^101^ Likewise, there is no clear evidence that the administration of prebiotics or lipopolysaccharides can prevent the development of allergies.^102^ Accordingly, there is currently no evidence to support the use of prebiotics or probiotics in the prevention of food allergies. However, research is underway to identify specific probiotics or prebiotics that affect allergy development.^99^

## Diagnosis and Management

**Figure 3:**
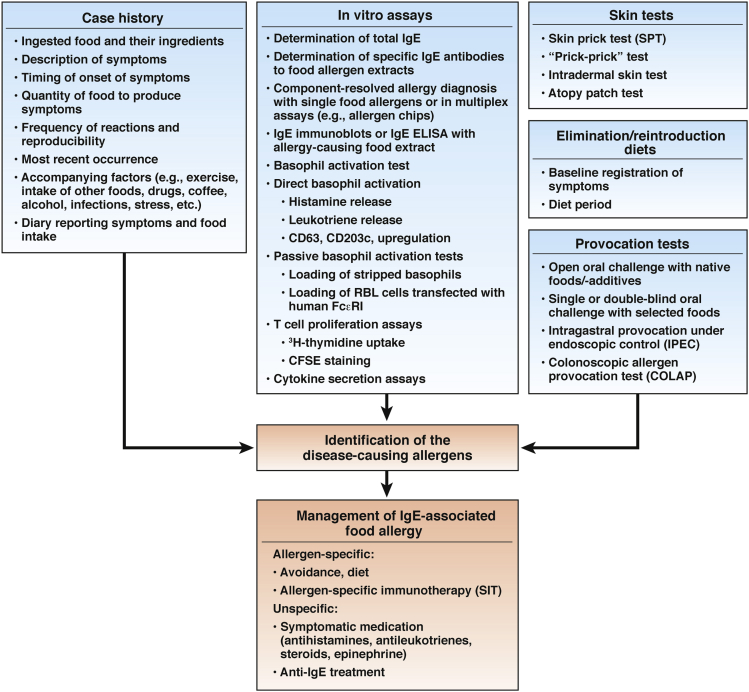
Diagnosis and management of IgE-associated food allergies. The diagnosis of a food allergy involves a case history and a demonstration of allergen-specific IgE production. Provocation tests and diets are used to identify disease-causing allergens. Based on this information, allergen-specific forms of treatment can be selected. This approach currently is being introduced into clinical practice. CFSE, carboxyfluorescein succinimidyl ester; ELISA, enzyme-linked immunosorbent assay; RBL, rat basophilic leukemia.

Once a patient is diagnosed with a food allergy, it becomes important to identify the allergen(s) that cause the disorder and determine if it is mediated by IgE. If so, treatments for IgE-associated allergies can be selected (Figure 3). In early studies of food allergies,^103^ diagnoses were based on careful analyses of case histories and diaries to document symptoms and offending foods.^6^ For more information on the diagnosis of food allergies, see Figure 3 and articles by Sicherer and Sampson,^3^ De Silva et al,^5^ and Boyce et al.^6^

Results from serologic and in vitro tests alone are not sufficient for the diagnosis of IgE-associated food allergies because the presence of allergen-specific IgE is not always associated with symptoms.^104^ This might be because certain antigens in food react with IgE but do not have allergenic activity. Furthermore, certain allergens easily are degraded and/or do not pass the epithelial barrier in sufficient quantities. For this reason, provocation tests often are useful and required to confirm IgE-associated food allergy. Skin tests are relatively easy to perform, but just as for IgE tests, results are not always associated with symptoms. Skin tests can detect IgE-mediated mast cell degranulation; these include the skin-prick test, prick-to-prick test, and intradermal test. Atopy patch tests can detect delayed-type reactions mediated by T-cell reactions.

Oral provocation tests are the most accurate in the diagnosis of clinically relevant IgE-associated food allergies once allergen-specific IgE has been detected. These involve placing patients on elimination diets, and re-introducing foods or providing an open oral challenge. A double-blind, placebo-controlled food challenge is the standard for antigen identification.^105^ However, the double-blind, placebo-controlled food challenge can induce severe reactions and requires careful planning and well-equipped clinical facilities.

Several provocation tests involve the application of mucosal allergens.^106^ Major advances in the diagnosis of food allergies and identification of disease-causing allergens include new in vitro multiplex allergy tests, which involve purified allergens.

IgE-associated food allergies are managed with allergen-specific treatments such as avoidance of the disease-causing allergens via diets that ensure balanced nutrition with the least possible effects on quality of life. The elimination diet is the most important and relevant long-term management strategy for food allergies.^5^ Once the offending food allergens have been identified the allergenic food must be avoided. For a summary of the management paths and guidelines for treatment, see the articles by Sicherer and Sampson,^3^ De Silva et al,^5^ and Boyce et al.^6^ For patients with a cow’s milk allergy, milk can be replaced with extensively hydrolyzed milk formulas.^107^

Allergen-specific immunotherapy (SIT) is currently the only allergen-specific and disease-modifying treatment that has long-term effects.^108^ SIT is used mainly to treat respiratory allergies, and less frequently to treat food allergies because standardized vaccines are not available. In the case of food allergies, SIT often is performed orally, by administration of the offending food instead of a vaccine.^68^, ^109^ Progress in the molecular characterization of food allergens will lead to the development of defined vaccines for the treatment of food allergies, as for respiratory allergens, which may become available in the future.^110^, ^111^, ^112^

Patients diagnosed with IgE-mediated food allergies can be given medications to reduce their symptoms.^4^, ^5^ These can be selected based on the involvement of IgE-mediated mast cell or basophil degranulation (antihistamines, antileukotrienes, epinephrine, anti-IgE), or T cells or eosinophil activation (steroids, anti-IL5) (Figure 3).^3^, ^6^ This procedure is beginning to enter clinical practice.

## Food Allergen Structure, Pathogenesis, and Diagnosis

**Figure 4:**
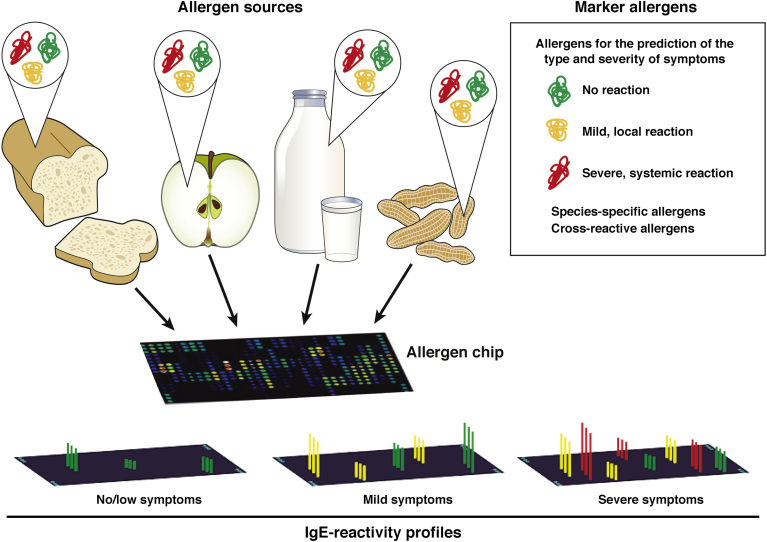
Component-resolved diagnosis of a food allergy. Different sources of food allergens contain several allergenic molecules (components); these can be produced as recombinant proteins or purified from natural sources. These allergens can be classified into IgE-reactive components (green), which are poor activators of inflammatory cells and therefore induce little or no clinical reactions; components that induce mild or mainly local symptoms (yellow); and components that often are associated with severe and systemic allergic reactions (red). Microarray technology can be used to determine reactivity profiles of patients. This process can be used to identify individual allergens that cause disease and foods to which patients are most likely to respond. The severity of reactions also can be predicted.

Since the 1980s, we have learned much about the structure and immunologic characteristics of allergens, and the clinical reactions they can cause.^110^ Researchers have produced recombinant allergens comprising repertoires of the most common antigens. Instead of ill-defined allergen extracts, which are prepared from the allergen sources (eg, wheat, apple, milk, or peanuts) (Figure 4) and consist of mixtures of various allergens and nonallergenic materials, pure allergen molecules are available for diagnosis and allergen-specific therapy. Purified recombinant allergens can be used to determine a patient’s IgE reactivity profile.

Many allergen sources contain antigens that have little or no clinical relevance because they are poor inducers of allergic reactions. These include IgE-reactive carbohydrate epitopes without allergenic activity,^113^ or molecules that induce only mild or local symptoms. Other molecules can induce severe systemic allergic reactions (Figure 4).

Marker allergens have been identified from the most common food allergen sources (eg, apple, peanut, milk, and wheat).^114^, ^115^, ^116^, ^117^, ^118^ Marker allergens are those found only in specific sources, and can be used to confirm sensitizations to these sources. Other allergens are present in different food sources. Patients who are sensitized to these can develop symptoms after ingestion of seemingly unrelated foods.

Individual allergen molecules are named and listed by the nomenclature committee of the World Health Organization and International Union of Immunological Societies.^119^ For an overview of the general biochemical characterization and features of allergens (see the article by Valenta^120^). Several databases also store information on allergens, including names, classifications, and characteristics (eg, http://www.allergen.org/; http://farrp.unl.edu/resources/farrp-databases; http://allergen.nihs.go.jp/ADFS/; http://www.allergome.org/; http://www.meduniwien.ac.at/allergens/allfam/; and http://www.allergenonline.org/).

Table 1 shows some important plant food and animal food allergen families. According to a recent classification, allergens from different sources can be grouped into families with similar biologic functions, primary structure, and immunologic cross-reactivity.^121^ A few examples for allergens from different sources that can be attributed to these structurally related allergen families are shown in Table 1.

**Table 1:** Plant Food and Animal Food Allergen Families.

Family	Function	Selected allergens (names/sources)
Plant food allergen families		
Prolamins	Seed storage proteins	Sec c 20/Rye; Tri a 19/wheat; Tri a 36/wheat
Nonspecific lipid transfer proteins	Involved in lipid transport, plant defense	Act d 10/Kiwi; Api g 2/celery; Ara h 9/peanut; Cas s 8/chestnut; Cor a 8/hazelnut; Jug r 3/walnut; Lyc e 3/tomato; Mus a 3/banana; Pru du 3/almond; Pru p 3 /peach; Tri a 14/wheat; Zea m 14/maize
2S albumins	Seed storage proteins	Ana o 3/cashew nut; Ara h 2/peanut; Ber e 1/Brazil nut; Fag e 2/buckwheat; Gly m 8/soybean; Jug r 1/walnut; Ses i 1/sesame; Sin a 1/mustard
Bet v 1 family	Pathogenesis-related proteins	Api g 1/celery; Ara h 8/peanut; Cor a 1/hazelnut; Dau c 1/carrot; Gly m 4/soybean; Mal d 1/apple; Pru p 1/peach
Cupin superfamily		
7S (vicilin-like) globulins	Seed storage proteins	Ana o 1/cashew nut; Ara h 1/peanut; Gly m 5/soybean; Jug r 2/walnut; Pis v 3/pistachio
11S (legumin-like) globulins	Seed storage proteins	Ana o 2/cashew nut; Ara h 3/peanut; Ber e 2/Brazil nut; Cor a 9/hazelnut; Gly m 6/soybean; Jug r 4/walnut; Pru du 6/almond
Cysteine protease C1 family	Cysteine proteases	Act d 1/kiwi; Gly m Bd 30K/soybean
Profilins	Actin-binding proteins	Act d 9/kiwi; Api g 4/celery; Ara h 5/peanut; Cuc m 2/melon; Dau c 4/carrot; Gly m 3/soybean; Lyc e 1/tomato; Mus a 1/banana; Ory s 12/rice; Pru av 4/cherry; Pru du 4/almond; Pru p 4/peach; Tri a 12/wheat
Animal food allergen families		
Tropomyosin family	Actin-binding proteins in muscle	Pen m 1/shrimp
Parvalbumin family	Muscle proteins, involved in muscle contraction	Cyp c 1/carp; Gad c 1/cod; Ran e 2/frog; Sal s 1/salmon; Seb m 1/redfish; Xip g 1/swordfish
Caseins	Mammalian milk proteins, form stable micellar complexes	Bos d 8–Bos d 12/cow’s milk
Transferrin family	Sulfur-rich ion-binding glycoproteins from milk and hen’s egg white	Bos d Lactoferrin/cow’s milk; Gal d 3/hen’s egg
Serpins	Serine protease inhibitors	Gal d 2/hen’s egg
Arginine kinases	Adenosine triphosphate: guanido phosphotransferases	Pen m 2/shrimp
Lipocalins	Carrier proteins	Bos d 5/cow’s milk
Lysozyme family	Enzymatic activity, lactose synthesis in milk	Bos d 4/cow’s milk; Gal d 4/hen’s egg
Ovomucoids	Kazal inhibitors, contain Kazal-type inhibitor repeats	Gal d 1/hen’s egg
Albumins	Serum albumins, transport proteins	Bos d 6/cow’s milk; Gal d 5/hen’s egg

NOTE. Biologic functions of the proteins and selected allergens from various food allergen sources are named according to the International Union of Immunological Societies allergen nomenclature.

The diagnosis of food and other allergies has transitioned from the identification of allergen sources without knowledge of the molecules that cause the symptoms, to the precise identification of allergy-inducing molecules. These processes are called “component-resolved allergy diagnosis” and “molecular allergy diagnosis.”^122^

Our ability to test a patient’s reactivity to a growing number of well-characterized allergen molecules has required the development of new diagnostic tests. We now can test small volumes of serum for IgE reactivity against multiple allergens simultaneously. Allergen chips containing micro-arrayed allergen molecules have been developed for this purpose^123^; they can be used to analyze serum samples for reactivity with a comprehensive set of molecules. This approach is ideal for analysis of children, or studies of differences or changes in allergic immune responses in large groups, such as birth cohorts.^124^ Based on component-resolved allergy diagnosis, the type and severity of symptoms can be predicted. Furthermore, the most relevant components can be identified to aid in the development of allergen-specific treatments and preventative strategies.^125^

## Allergen-Specific Prevention and Therapy

**Figure 5:**
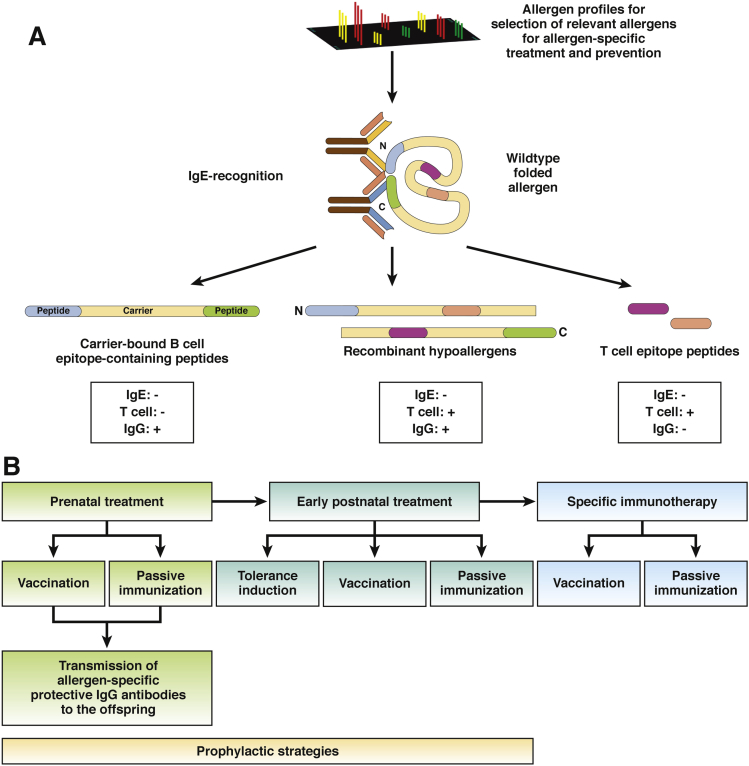
Allergen-specific forms of prophylaxis and treatment. Multiplex allergen systems can be used to identify disease-relevant food allergens in populations. (*A*) Based on the mapping of antigen epitopes recognized by patients’ IgE and T cells, 4 molecular approaches are being developed for prophylaxis and treatment. These are as follows: recombinant wild-type allergens, carrier-bound B-cell epitope–containing peptides (which do not react with IgE, have reduced allergen-specific epitopes recognized by T cells, and induce allergen-specific IgG), recombinant hypoallergens (which have reduced reactivity with IgE and fewer epitopes that interact with T cells, and induce allergen-specific IgG), and peptide epitopes that interact with T cells (but do not react with IgE or induce allergen-specific IgG). (*B*) Allergen-specific treatment can be prophylactic (prenatal or early postnatal) or be given after sensitization has taken place (specific immunotherapy). Active vaccination, passive immunization with allergen-specific antibodies, and tolerance induction are options.

A major limitation of SIT is the difficulty in preparation of effective and safe vaccines from natural allergen sources.^126^ However, based on the knowledge of the structure of the disease-causing allergens, it has become possible to produce new forms of allergy vaccines based on purified allergen molecules (Figure 5).^110^ Clinical trials have shown the efficacy of immunotherapies that include recombinant allergens in the wild-type, folded forms.^127^ Vaccines can be developed based on these allergens, under standardized conditions.

However, wild-type allergens still contain epitopes that activate IgE and T cells, and therefore might induce allergic reactions in patients. Three approaches, based on modified allergens, have been developed to make allergy vaccines more safe, effective, and convenient. These allow for selective targeting of different facets of the allergic immune response. They include synthetic allergen-derived peptides that contain allergen-specific T-cell epitopes without IgE reactivity.^128^ Because of their small size, peptide vaccines can induce T-cell tolerance without allergen-specific IgG responses. Recombinant hypoallergenic allergen derivatives are characterized by strongly reduced IgE reactivity, and contain allergen-specific T-cell epitopes. After internalization, they can induce allergen-specific IgG responses.^129^ Carrier-bound peptides that contain B-cell epitopes are fusion proteins that consist of an allergen-unrelated carrier protein and nonallergenic peptides from the IgE binding sites of allergens. They lack IgE reactivity and most allergen-specific T-cell epitopes, but can induce allergen-specific IgG antibodies.^130^

These approaches are in immunotherapy trials for patients with respiratory allergies. Results from clinical studies have indicated that the induction of allergen-specific IgG that blocks the interaction between allergens and IgE are necessary for immunotherapy of respiratory and food allergies.^66^, ^108^ It therefore is conceivable that food allergies can be treated in a similar manner as respiratory allergies—not only by oral SIT but also by vaccination protocols. In fact, a vaccine for fish allergies, based on a recombinant hypoallergenic derivative of the major fish allergen (Cyp c 1), is being evaluated in a phase 2 immunotherapy trial in a European Union–funded research program.^112^, ^131^, ^132^, ^133^

Food allergies also might be prevented with prenatal or early postnatal strategies to prevent allergic sensitization. Studies of animal models have shown that vaccination of pregnant mice with molecules that induce allergen-specific IgG, or administration of allergen-specific IgG, prevented allergic sensitization in offspring.^134^, ^135^ There is evidence that post-natal administration of hydrolyzed milk that contains allergen-derived peptides can induce tolerance.^136^ However, a recent study in which children at risk for celiac disease were given gluten as infants showed no effects.^137^ Research programs are underway to determine when and how best to expose infants to potential allergens (eg, peanut allergens) to avoid sensitization and/or induce tolerance.^138^

In addition to prenatal approaches, strategies also might be developed to prevent allergy based on early vaccination or administration of allergen-specific IgG shortly after birth.^139^ In addition to subcutaneous administration, immunotherapy for food allergy can be given by sublingual, oral, or epitcutaneous delivery.^140^ Sublingual therapy has been used for therapeutic vaccination but recently was found to be safe and induce tolerance in children with IgE sensitization but without allergic symptoms.^141^ Clinical studies of patients with egg or peanut allergies found that oral immunotherapy not only is effective for treatment,^66^ but also induces sustained protection, for up to 5 years.^142^ Various forms of oral immunotherapy currently are used in different countries.

We need to identify disease-relevant allergens as well as windows for early intervention if we are to develop preventive allergen-specific treatments. Studies are underway in birth cohorts. Because of the higher prevalence of respiratory allergies than food allergies, it is likely that first clinical prevention studies will be performed for respiratory allergens. However, findings should be applicable to food allergies.

Specific immunotherapy of sensitized patients with recombinant and synthetic vaccines is most advanced for respiratory allergies, but approaches applied to respiratory allergens will be used to help develop defined vaccines for food allergy. Hypoallergenic allergen derivatives already have been made for several important food allergens and were evaluated in vitro and in animal models^143^; a vaccine for fish allergies has been administered safely to patients in a clinical trial.^133^ We therefore can expect profound advances in sublingual immunotherapy for food allergies through recombinant allergen-based vaccines in the near future.

## Figures and Tables

**Supplementary Table 1:** Mechanisms and Clinical Manifestations of IgE-Associated Food allergy

Organ system	Clinical manifestations	Immunopathology	Features	Age and natural course
Skin	Urticaria, angioedema Flush, pruritus Oral allergy syndrome (local itching and tingling and/or edema of lips, tongue, palate and pharynx)	IgE-mediated mast cell/basophil degranulation	Acute onset after food ingestion (minutes-hours)	In infants and adults, may resolve with age Appears mainly in adults with established pollen allergies, long-lived, boosted by pollen contact
	Contact urticaria		After direct skin contact	In infants and adults

Atopic dermatitis Protein contact dermatitis	T cell-mediated (with or without involvement of IgE)	Delayed type reaction > 24 hours after food ingestion	In infants and adults

Respiratory tract	Laryngeal and/or pharyngeal edema Hoarseness, cough	IgE-mediated mast cell/basophil degranulation	Acute onset after food ingestion (minutes-hours)	In infants and adults

Rhinoconjunctivitis Bronchial asthma	T cell-mediated (with or without involvement of IgE)	Delayed type reaction > 24 hours after food ingestion	In infants and adults Baker’s asthma in adults

Gastrointestinal tract	Colitis Diarrhea Gastroenteritis Anorexia Nausea, vomiting Abdominal pain Flatulence Abdominal distension	IgE-mediated mast cell/basophil degranulation	Acute onset after food ingestion (minutes-hours)	In infants and adults

	Gastroenteritis	T cell-mediated (with or without involvement of IgE)	Delayed type reaction > 24 hours after food ingestion, increased pro-inflammatory cytokine responses	In infants and adults, may resolve with age

Eosinophilic gastroenteritis Eosinophilic esophagitis	Eosinophilic gastroenteritis	Eosinophil-activation by cytokines	In infants and adults

Cardiovascular system	Tachycardia Hypotension Vascular collapse Anaphylactic shock Cardiac dysrhythmia	IgE-mediated mast cell/basophil degranulation	Acute onset after food ingestion (minutes-hours)	In infants and adults

Nervous system	Irritability Anxiety	IgE-mediated mast cell/basophil degranulation?	Acute onset after food ingestion (minutes-hours)	In infants and adults
	Confusion	and/or	and/or	
	Loss of consciousness	T cell-mediated (with or without involvement of IgE)?	Delayed type reaction > 24 hours after food ingestion	
